# Effects of Annealing Temperature on Properties of Ti-Ga–Doped ZnO Films Deposited on Flexible Substrates

**DOI:** 10.3390/nano5041831

**Published:** 2015-11-03

**Authors:** Tao-Hsing Chen, Ting-You Chen

**Affiliations:** Department of Mechanical Engineering, National Kaohsiung University of Applied Sciences, Kaohsiung City 807, Taiwan; E-Mail: stars@pchome.com.tw

**Keywords:** annealing temperature effect, Ti-Ga, flexible substrate, polyimide

## Abstract

An investigation is performed into the optical, electrical, and microstructural properties of Ti-Ga–doped ZnO films deposited on polyimide (PI) flexible substrates and then annealed at temperatures of 300 °C, 400 °C, and 450 °C, respectively. The X-ray diffraction (XRD) analysis results show that all of the films have a strong (002) Ga doped ZnO (GZO) preferential orientation. As the annealing temperature is increased to 400 °C, the optical transmittance increases and the electrical resistivity decreases. However, as the temperature is further increased to 450 °C, the transmittance reduces and the resistivity increases due to a carbonization of the PI substrate. Finally, the crystallinity of the ZnO film improves with an increasing annealing temperature only up to 400 °C and is accompanied by a smaller crystallite size and a lower surface roughness.

## 1. Introduction

Transparent conducting thin films deposited on glass substrates are widely used throughout the semiconductor and electronics industries. However, glass is heavy and brittle, and is thus unsuitable for emerging flexible electronic devices, such as smart cards, electronic maps, flat panel displays, and so on [1,2]. Consequently, the feasibility of utilizing polymer materials as a substrate material has attracted increasing interest in recent years [3,4]. Among the various conducting thin film materials available, ZnO, doped with elements such as Al [5], Ga [6], In [7], and Mo [8], has received particular attention due to its excellent optical and electrical properties. Ti-Ga co-doped ZnO thin films on glass substrates have also been widely investigated in recent years due to their high visible transparency and electrical conductivity [9,10]. However, the properties of Ti-Ga–doped ZnO films on flexible substrates have attracted little interest in the literature thus far.

Various methods exist for the deposition of thin-film structures on glass or polymer substrates, including spray pyrolysis [11], chemical vapor deposition [12], sol-gel processing [13], and sputtering [14]. Many studies have shown that films deposited using radio-frequency (RF) magnetron sputtering have good crystallinity and microstructural uniformity even at low substrate temperatures [9,15,16,17]. However, the microstructure and properties (optical and electrical) of these films vary significantly with the discharge power, working gas pressure, distance between the target and substrate, and post-deposition annealing temperature.

Accordingly, in the present study, Ti-Ga–doped ZnO films are deposited on polyimide (PI) substrates using the RF magnetron sputtering method. The optical, electrical, and microstructural properties of the films are then investigated following annealing at temperatures of 300 °C, 400 °C, and 450 °C, respectively.

## 2. Experimental Procedure

Ti-Ga–doped ZnO thin films were deposited on PI substrates with dimensions of 2.0 cm × 4.0 cm × 200 μm. The RF sputtering system consisted of a Ga:ZnO alloy target with a RF power supply and a Ti metallic target (purity 99.999%) with a Directly Current (DC) power supply. Both targets measured 2 inches by 0.50 cm (diameter × thickness). The Ga:ZnO target was sintered from Ga doped ZnO (GZO) pellets containing ZnO (purity 99.2%) doped with 3% Ga_2_O_3_ (99.9%). Prior to sputtering, the substrates were cleaned in acetone and methanol in an ultrasonic bath and then dried under a nitrogen gas flow. In the sputtering process, the distance between the targets and the substrate was fixed at 6 cm and the flow rate and pressure of the Argon working gas were set as 15 cm^3^/min and 1.33 × 10^−3^ Pa, respectively. The sputtering process commenced by depositing a GZO film on the PI substrate using a sputtering power and deposition rate of 100 W and 3.33 nm/min, respectively. A thin Ti layer was then sputtered on the GZO film with a sputtering power of 60 W and a deposition rate of 0.85 nm/min. The as-deposited specimens were then annealed for 20 min in a vacuum annealing furnace at temperatures of 300 °C, 400 °C, and 450 °C, respectively.

The microstructural characteristics of the as-sputtered and annealed films were investigated by X-ray diffraction (XRD, SIEMENS D5000). In addition, the electrical resistivity was evaluated using a Hall Effect measurement system (AHM-800B). The transmittance spectra were measured at wavelengths of 300~800 nm using a UV-VIS spectrophotometer (Hitachi U-4001). Finally, the surface morphology was examined using a scanning electron microscope (SEM, Philip’s XL-40 FEG) and an atomic force microscope (AFM) operated in a contact mode.

## 3. Results and Discussions

### 3.1. Thickness and Structural Analysis of Ti-Ga–Doped ZnO Films

**Figure 1:**
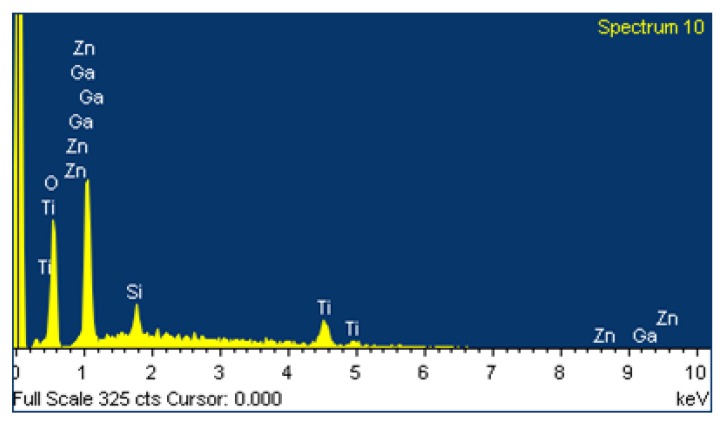
Energy dispersive X-ray (EDX) analysis results for as-deposited Ti-Ga–doped ZnO film.

The thickness of the GZO and Ti layers was calculated based on the deposition time (30 min) and deposition rate. The thickness of the GZO layer was found to vary between 100 and 120 nm, while that of the Ti layer varied between 50 and 60 nm. The chemical composition of the as-deposited Ti-Ga–doped ZnO film was examined by means of energy dispersive X-ray (EDX) spectroscopy. As expected, the film consists mainly of Zn, Ga, and Ti elements (Figure 1).

**Figure 2:**
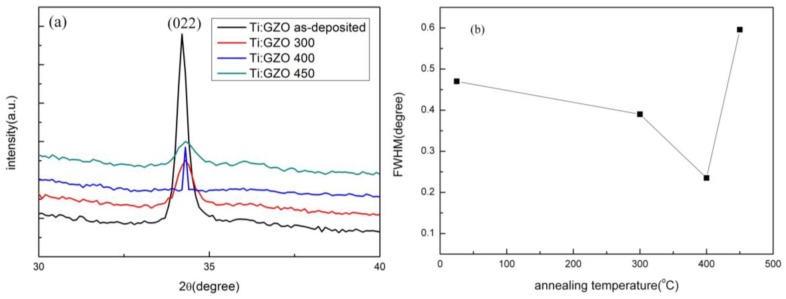
(**a**) X-ray diffraction (XRD) patterns; (**b**) Full width at half maximum (FWHM) values of as-deposited and annealed Ti-Ga–doped ZnO thin films as a function of annealing temperature.

Figure 2a shows the XRD spectra of the as-deposited and annealed Ti-Ga–doped ZnO films. It is seen that all the films have a strong diffraction peak located at around 2θ = 34.3°. The position of this peak is consistent with that of standard ZnO crystal (34.45°). Hence, it is inferred that the GZO thin films have the structural characteristics of hexagonal ZnO wurtzite. The absence of Ga and Ti peaks in the XRD scans suggests that Ga and Ti are present only in very small quantities and are confined to the non-crystalline region of the annealed films at the grain boundaries [18,19,20]. It is observed that the intensity of the diffraction peak increases as the annealing temperature is increased to 400 °C, but decreases as the temperature is further increased to 450 °C. In other words, the microstructure of the Ti-Ga–doped ZnO films becomes increasingly crystalline under higher temperatures (at 400 °C). Figure 2b shows the full width at half maximum (FWHM) values of the as-deposited and annealed Ti-Ga–doped ZnO films. It is seen that the FWHM decreases as the annealing temperature increases from 300 to 400 °C, but increases at the highest annealing temperature of 450 °C. In other words, the maximum crystallinity occurs in the film annealed at 400 °C. Compared to Ti co-doped GZO films deposited on glass substrates [21], the diffraction peak intensity of the present films is rather weak. Moreover, the FWHM values are somewhat larger.

### 3.2. Optical and Electrical Properties of Ti-Ga–Doped ZnO Films

**Figure 3:**
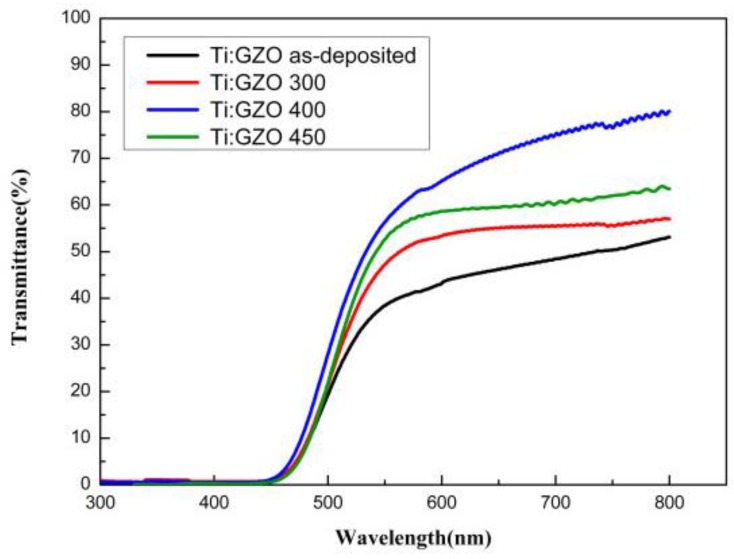
Optical transmittance of as-deposited and annealed Ti-Ga–doped ZnO thin films.

Figure 3 shows the transmission spectra of the as-deposited and annealed Ti-Ga–doped ZnO films over the wavelength range of 300–800 nm. It is seen that the as-deposited film has a low transparency of just 60% in the visible region and has a sharp absorption edge at ~380 nm. For the annealed ZnO films, the transmittance increases to approximately 80% as the annealing temperature is increased from 300–400 °C. In other words, the transformation of the ZnO film to a crystalline structure prompts an improvement in the optical transmittance. However, at a higher temperature of 450 °C, the transmittance reduces to around 75%. The PI substrate has a maximum endurance temperature of approximately ~500 °C. Thus, it is speculated that the reduced optical transmittance of the ZnO film at higher annealing temperatures is due to thermally induced carbonization of the underlying PI substrate.

**Figure 4:**
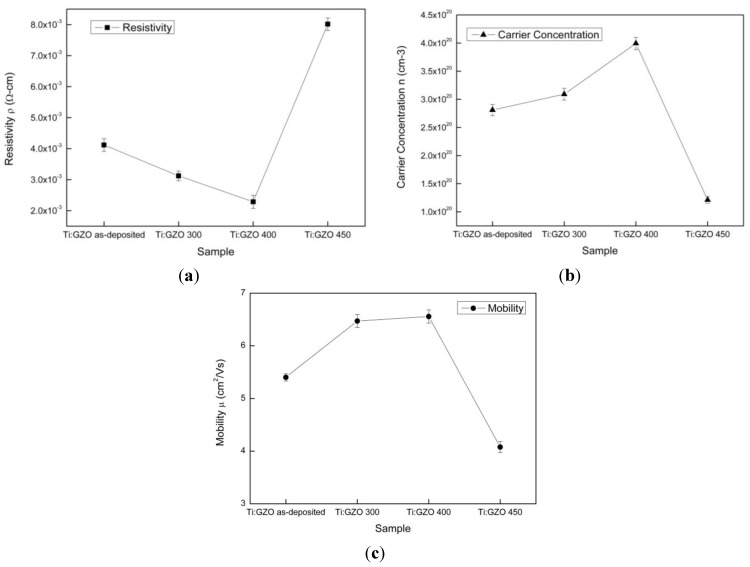
(**a**) Resistivity; (**b**) Carrier concentration; and (**c**) Hall mobility of as-deposited and annealed Ti-Ga–doped ZnO thin films.

Figure 4a–c show the resistivity, carrier concentration, and Hall mobility properties of the as-deposited and annealed films. As shown in Figure 4a, the resistivity reduces from 4 × 10^−3^ to 2.2 × 10^−3^ Ω-cm as the annealing temperature increases from 25 to 400 °C. The lower resistivity stems from the improved crystallinity of the Ti-Ga ZnO film structure, a lower scattering of the charge carriers at the grain boundary, the increased substitutional doping, and a reduction in the number of interstitial atoms. The reduced resistivity also suggests an improved diffusion of the Ti atoms into the Ga-doped ZnO film under higher temperatures. For the specimen annealed at the highest temperature of 450 °C, the resistivity increases to approximately 7.9 × 10^−3^ Ω-cm. The increased resistivity can be attributed to the carbonization of the PI substrate. Figure 4b,c shows that the carrier concentration and Hall mobility increase as the annealing temperature increases from 25 to 400 °C. In other words, a higher annealing temperature prompts a greater number of free carrier electrons and improves the conductivity. However, both properties reduce at a higher annealing temperature of 450 °C due to carbonization of the PI layer.

### 3.3. AFM and SEM Surface Observations

**Figure 5:**
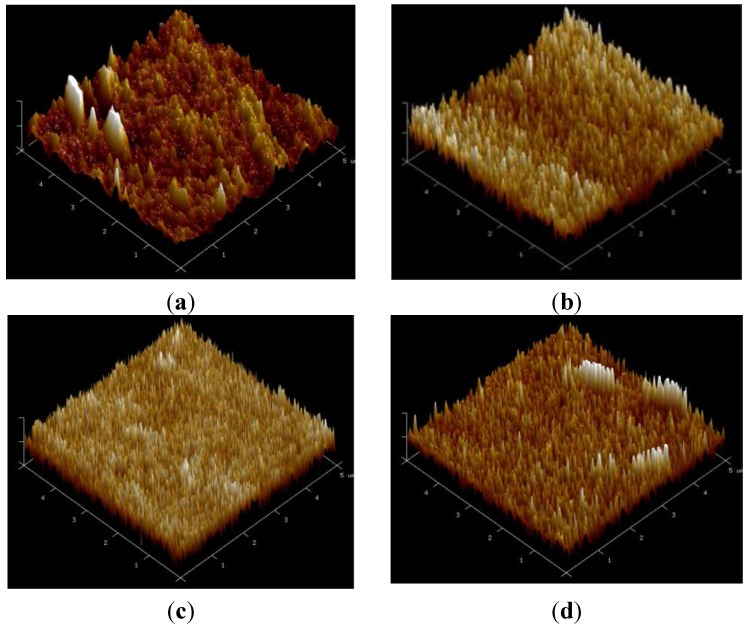
Three-dimensional (3D) atomic force microscope (AFM) images of Ti-Ga–doped ZnO thin films in (**a**) as-deposited condition; and following annealing at: (**b**) 300 °C; (**c**) 400 °C; and (**d**) 450 °C.

Figure 5a–d show the AFM morphologies of the as-deposited and annealed Ti-Ga–doped ZnO films. The images show that the crystallinity of the films improves with an increasing annealing temperature up to 400 °C and is accompanied by a smaller crystallite size. The smaller crystallite size leads to an improved smoothness due to the formation of a more dense surface morphology.

**Figure 6:**
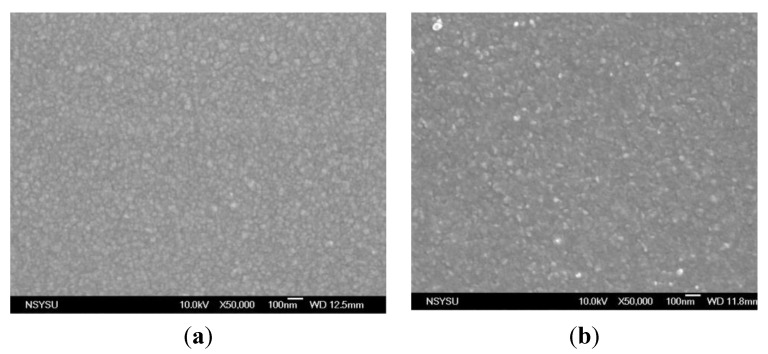

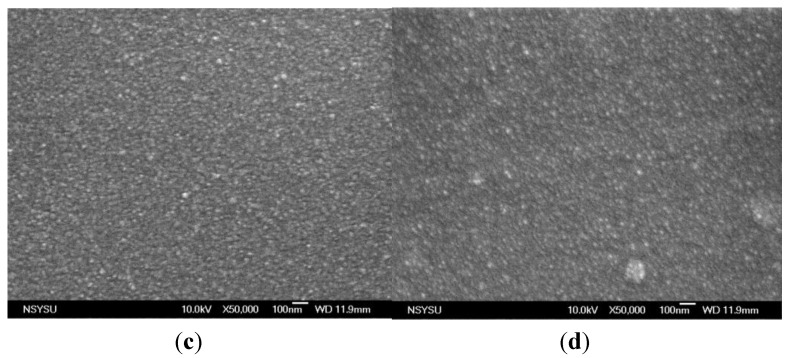
Scanning electron microscope (SEM) images of Ti-Ga–doped ZnO films in (**a**) as-deposited condition; and following annealing at: (**b**) 300 °C; (**c**) 400 °C; and (**d**) 450 °C.

Figure 6a–d present SEM images of the various Ti-Ga–doped ZnO films. The surface morphology of the as-deposited film has a pebble-like appearance with an average grain size of around 70 nm (Figure 6a). However, an inspection of Figure 6b–d shows that the average grain size decreases to approximately 30 nm as the annealing temperature is increased to 450 °C. This finding is to be expected since a higher annealing temperature increases the average energy of the bombarding ions on the growing film, thereby enhancing the atom mobility on the surface. Moreover, an increasing annealing temperature also increases the diffusion rate, thereby allowing a more perfect Ti-Ga–doped ZnO film crystal to be obtained.

## 4. Conclusions

This study has investigated the effects of the annealing temperature (300~450 °C) on the optical, electrical, and microstructural properties of Ti-Ga–doped ZnO films deposited on PI flexible substrates. The XRD analysis results have shown that the as-deposited and annealed films have a hexagonal structure with a preferred orientation of the c-axis perpendicular to the substrate. The transmittance of the ZnO films increases to approximately 80% as the annealing temperature is increased from 300~400 °C due to an improved crystallinity of the ZnO microstructure. However, the transmittance reduces to 75% as the annealing temperature is further increased to 450 °C due to a carbonization of the PI substrate. The electrical resistivity of the ZnO films falls to a minimum value of approximately 2.2 × 10^−3^ Ω-cm at an annealing temperature of 400 °C. However, the resistivity, carrier concentration, and Hall mobility are all degraded at a higher annealing temperature of 450 °C due to substrate damage. The AFM and SEM surface observations have shown that a higher annealing temperature prompts an improved crystallinity of the doped ZnO microstructure, a smaller grain size, and a reduced surface roughness. In general, the quality of the Ti-Ga–doped ZnO films deposited on PI substrates is somewhat poorer than that of equivalent films deposited on glass substrates. However, the present results suggest that given an appropriate control of the post-deposition annealing temperature, Ti-Ga–doped ZnO films deposited on PI substrates represent a feasible solution for emerging flexible electronics applications.
